# Haemoglobin Concentration and Cognitive Ability in the Aberdeen Children of the 1950s

**DOI:** 10.7759/cureus.21806

**Published:** 2022-02-01

**Authors:** Mohammed T Bashir, Chris McNeil, Usman Rasul, Alison Murray

**Affiliations:** 1 Aberdeen Biomedical Imaging Centre, University of Aberdeen, Aberdeen, GBR; 2 Department of Obstetrics and Gynaecology, Glasgow Royal Infirmary, Glasgow, GBR

**Keywords:** dementia, ageing, cognition, anaemia, haemoglobins

## Abstract

Introduction

Haemoglobin concentrations decrease with age. Abnormally low and high haemoglobin concentrations are associated with reduced cognition; however, the evidence for these associations in cohort data is limited. This study aims to assess the relationship between haemoglobin concentration and cognition in a well-characterised cohort of older adults.

Methods

Two hundred and fifty-two healthy participants were drawn from the Aberdeen Children of the 1950s cohort, aged between 59 to 65 years. Participants underwent cognitive tests of processing speed, memory, verbal and nonverbal reasoning, and language ability and these were used to construct a global cognitive score, *g*, using principal component analysis. Haemoglobin concentration in the blood was measured concurrently. Hierarchical multiple regression models were constructed assessing the relationship between haemoglobin concentration and each cognitive measure and these were corrected for age, sex, education, C-reactive protein, hypertension, and body mass index.

Results

Significant linear association between haemoglobin concentration and nonverbal reasoning demonstrated that low haemoglobin levels are associated with lower scores. A quadratic relationship was found for haemoglobin concentration and immediate memory scores in which low and high haemoglobin levels were associated with lower scores.

Conclusions

Haemoglobin concentration was found to have a significant linear association with nonverbal reasoning scores and a significant quadratic association with memory scores. The results from this study help to understand the association between haemoglobin and different aspects of cognition.

## Introduction

Mean haemoglobin concentration progressively declines with age. Anaemia, defined by the World Health Organisation as a blood haemoglobin concentration of less than 12g/dl in females and less than 13g/dl in males, is common in older adults, about 24% of whom are anaemic worldwide [1]. Common complications of this decrease in haemoglobin concentration include fatigue and weakness with a noticeable functional disability as well as poorer physical performance [2].

Recent studies in older people have linked anaemia with cognitive impairment demonstrating a reduction in executive function, memory and mental status, and have labelled anaemia as an important risk factor for the development of dementia [3]. The mechanisms underlying this link are unclear; however, it is reasonable to assume that the resultant hypoxia limits brain energy metabolism and may lead to functional and structural impairment. Several studies have demonstrated an association between anaemia and reduced brain volumes [4-5] as well as an increase in brain pathologies [6]. There has also been a reverse causal relationship suggested in which impaired cognition may be the cause of anaemia, potentially through poor diet and/or forgetting to eat [3].

Evidence that anaemia, or variations in haemoglobin concentrations, have a detrimental effect on cognition from cohort data is limited. Epidemiologic analysis of older adults shows that anaemia, in a German cohort, increased the risk of mild cognitive impairment [7] and low haemoglobin in a Chinese cohort was linked with reduced cognition both cross-sectionally and after a two-year interval [8]. Other longitudinal studies have shown a U-shaped relationship, with low and high haemoglobin levels predicting cognitive decline and Alzheimer’s Disease risk [9]. Clearly, the level of haemoglobin can potentially be a marker or a cause of low cognition and must be considered when predicting cognitive performance in older people.

This study aims to better understand the importance of abnormal haemoglobin levels for cognitive function and brain health in nondemented normal older adults.

## Materials and methods

Sample

The sample was drawn from the Aberdeen Children of the 1950s (ACONF), a well-characterized, healthy cohort of people born between 1950-56 and recruited into a study of early life predictors of health in the early 2000s [10]. Since 2015, 252 ACONF participants, aged between 59 and 65 years, entered the Stratifying Resilience and Depression Longitudinally (STRADL) study. Participants were invited into the STRADL study based on their membership of ACONF and the Generation Scotland: Scottish Family Health Study (GS: SFHS). Participants were excluded from the study if they had any contraindications to magnetic resonance imaging such as metallic implants [11]. All components of STRADL received formal, national ethical approval from the National Health Service (NHS) Tayside committee on research ethics (reference 14/SS/0039).

Blood sampling and analysis

Participants underwent blood sampling by venepuncture with a butterfly needle set. Amongst the blood samples taken, a vacutainer containing ethylenediamine tetraacetic acid (EDTA) was collected and analysed for a full blood count (FBC) by a Beckman analyzer (Beckman Coulter Inc., Brea, California, United States). This provided data for haemoglobin concentration, white cell count, platelet count, haematocrit, mean cell volume (MCV), mean corpuscular haemoglobin, neutrophil count, lymphocyte count, monocyte count, eosinophil count, basophil count, and leucocyte count. Mean corpuscular haemoglobin concentration (MCHC) was also calculated. Whole blood was collected into a vacutainer containing a clot activator gel. Following serum separation, the concentration of C-reactive protein (CRP) was determined. We defined anaemia as a haemoglobin level of less than 12g/dl in females and less than 13g/dl in males in accordance with World Health Organization parameters [12]. We defined high haemoglobin as greater than 15.5g/dl in females and greater than 17.5g/dl in males based on values given by Mayo Clinic [13].

Blood pressure (BP)

The participant was asked to sit for five minutes at room temperature. BP was then measured twice using a calibrated Omron sphygmomanometer (Omron Corp., Kyoto, Japan) and an appropriately sized cuff. The mean of the readings was taken. A participant was considered hypertensive if they had a diastolic BP of greater than 90 mmHg or a systolic BP of greater than 140 mmHg [14].

Other confounding variables

Patients’ height and weight were recorded and body mass index (BMI) was derived by dividing weight (kilograms) by height (m^2^). Data on educational attainment were obtained via survey and divided into three ordinal levels: completion of up to compulsory education, completion of up to college education, and completion of higher education.

Cognitive and health assessment

Participants underwent a variety of cognitive tests, which assessed different cognitive domains. Both immediate and delayed memory was tested using the Wechsler Memory Scale, the 4th edition (WMS-IV) - Logical Memory (LM) I and LM II, respectively [15]. Processing speed was assessed using the Digit Symbol (DS) subsection of the Wechsler Adult Intelligence Scale [15]. The Mill Hill Vocabulary (MHV) test was used to assess language ability [15]. Nonverbal reasoning was assessed using Raven's Standard Progressive Matrices (RPM) and the Controlled Oral Word Association task was used as a measure of verbal fluency [15].

Derivation of a general cognitive score (*g*)

We constructed a global cognitive score using principal component analysis of total scores from all cognitive tests (processing speed, immediate and delayed memory, verbal fluency, nonverbal reasoning, and language ability). We examined the scree plot and extracted the first unrotated component, which had an Eigenvalue of 2.48 and contained 41.4% of the total variance. This factor correlated most strongly with immediate and delayed memory scores, with correlation coefficients of 0.798 and 0.984 respectively, and least with language scores with a correlation coefficient of 0.272. We then converted the data into IQ-like scores by multiplying *g* by 15 and adding 100. This generated data with a mean of 100 and a standard deviation of 15, defined as IQ-like [16].

Statistical analysis

First, the relationships between haemoglobin and confounding variables: age, sex, education, BMI, hypertension, and CRP were assessed by simple linear regression.

To investigate any relationship between haemoglobin and cognition, haemoglobin concentration was plotted on a scatter chart against cognitive test scores. Further analysis was then conducted with simple linear regression models and we then constructed hierarchical multiple regression models. For our first model, we included nonvariable participant factors: age and sex. For our second model, we added variable risk factors: BMI and hypertension. Our third model included educational attainment and our fourth model added CRP. We repeated this analysis with the inclusion of a haemoglobin*haemoglobin variable to investigate a quadratic relationship.

Statistical significance was fixed at <0.05 with 95% confidence intervals and data analysis was conducted using IBM SPSS Statistics for Windows, Version 25.0 (Released 207. IBM Corp., Armonk, New York).

## Results

Participant characteristics

Table 1 displays the demographic data of our sample. Our analysis included 252 participants, 138 females and 114 males, from the STRADL study, aged between 59 and 65 years with a mean of 62 years. The mean haemoglobin concentration of the total sample was 14.5±1.2g/dL with means of 15.2g/dL and 13.9g/dL in males and females respectively. The mean BMI for our participants was 27.9 ± 5.6kg/m^2^. We identified 134 participants who were hypertensive and 188 participants were educated to at least a college level, 112 of whom received higher education (not shown).

**Table 1:** Summary of sample data P-value refers to two-way Student's t-test except in † where a chi-squared test was performed.

Variable		All Participants	Male	Female	P value
Sex (m/f)		252	114	138	.33^†^
Age (years)		62.3±1.5	62.4±1.5	62.2±1.6	.36
Haemoglobin (g/dL)		14.5±1.2	15.2±1.1	13.9±1.0	.00
Body Mass Index (Kg/m^2^)		27.8±5.6	27.6±4.3	27.9±6.5	.73
Digit Symbol		64.92±13.1	63.3±13.7	66.2±12.5	.09
Immediate Memory		14.9±3.5	14.0±3.7	15.7±3.2	.00
Delayed Memory		13.9±3.8	12.8±3.8	14.8±3.5	.00
Verbal reasoning		40.9±11.2	39.2±11.1	42.2±11.2	.04
Nonverbal Reasoning		8.5±2.5	8.6±2.6	8.3±2.3	.35
Language		31.7±4.1	31.8±4.6	31.6±3.7	.67
g		100±15	96.6±15.4	102.0±13.8	.01
Anaemia Status (n)	anaemic	7	2	5	NA
	normal	237	111	126	
	high	8	1	7	

For cognitive tests, female participants had significantly higher scores than males respectively in both immediate (15.7±3.2 and 14.9±3.5) and delayed memory (14.8±3.5 and 12.8±3.8), verbal reasoning (42.2±11.2 and 39.2±11.1), and for *g*, our derived global cognitive score (102.0±13.8 and 96.6±15.4). There was no significant difference between females and males in digit symbol, nonverbal reasoning and language measures.

Haemoglobin and demographic factors

Table 2 displays bivariate correlations between haemoglobin and demographic variables. There was no relationship between age and haemoglobin concentration in our sample. As expected, sex had a significant association with haemoglobin and was higher in males than females (P<0.001). Hypertension (P<0.001), BMI (P=0.024), and CRP (P=0.016) were found to be significantly associated with haemoglobin concentrations and education showed no association (P=0.986).

**Table 2:** Summary of association between haemoglobin and confounding variables P-value refers to simple linear regression CRP: C-reactive protein

Variable	R-Squared	P-value
Age	-.003	.621
Sex	.273	.000
Hypertension	.055	.000
BMI	.017	.024
CRP	.019	.016
Education	-.004	.986

Haemoglobin and cognition

Table 3 displays results from our simple regression models predicting cognitive scores with haemoglobin (g/dL). Haemoglobin was found to have a significant inverse relationship with delayed memory scores (p=0.028) and positive relationship with nonverbal reasoning scores, which trended towards significance (p=0.055). No significant associations were present for other cognitive tests nor for *g*. 

**Table 3:** Univariate association between haemoglobin concentration and cognitive tests P-value refers to two-way simple linear regression

Variable	Standardised beta	P value
Digit Symbol	-0.113	0.078
Immediate Memory	-0.116	0.070
Delayed Memory	-0.141	0.028
Verbal reasoning	-0.103	0.111
Nonverbal Reasoning	0.013	0.055
Language	0.040	0.537
g	-0.087	0.180

Table 4 is an example of how our hierarchical multiple linear regression models were developed. In this example, the relationship was initially nonsignificant (P=0.081) but became more significant with each iteration of the model and a similar trend was seen for the other cognitive tests. 

**Table 4:** Hierarchical multiple linear regression model: matrix reasoning P-value refers to multiple linear regression Haem: haemoglobin; CRP: C-reactive protein

Model 1				Model 2 (+variable risk factors)				Model 3 (+education)				Model 4 (+CRP)		
Variable	Statistic			Variable	Statistic			Variable	Statistic			Variable	Statistic	
	Std Beta	P-value			Std Beta	P-value			Std Beta	P value			Std Beta	P value
Haem	0.131	0.081		Haem	0.179	0.021		Haem	0.178	0.015		Haem	0.187	0.013
Age	0.064	0.319		Age	0.055	0.392		Age	0.057	0.349		Age	0.059	0.336
Sex	-0.052	0.888		Sex	-0.023	0.757		Sex	-0.027	0.700		Sex	-0.032	0.665
				Hypertension	-0.095	0.149		Hypertension	-0.090	0.150		Hypertension	-0.089	0.156
				BMI	-0.127	0.053		BMI	-0.090	0.113		BMI	-0.106	0.094
								Education	0.311	0.000		Education	0.317	0.000
												CRP	0.040	0.526
R^2^	0.019			0.045				0.141				0.142		
Adjusted R^2^	0.007			0.025				0.119				0.117		

Table 5 shows the results of our final hierarchical regression models investigating the linear (Model A) and quadratic (Model B) relationship between haemoglobin and cognition. After correcting for all relevant confounding variables, haemoglobin was found to have a significant positive association with nonverbal reasoning scores (P=0.013). A significant quadratic relationship was found for immediate memory scores (P=0.034).

**Table 5:** Results from linear and quadratic multiple hierarchical regression models Standardised beta values are reported; in parentheses, standard error and P-values are indicated. Model A: from a regression model containing: haemoglobin, age, sex, education, C-reactive protein, hypertension, and BMI Model B: model A with the addition of a haemoglobin*haemoglobin variable

Cognitive Outcome	Parameter estimate	
	Model A (haemoglobin)	Model B (haemoglobin*haemoglobin)
Digit symbol	- 0.066 (0.087, 0.488)	- 0.310 (0.004, 0.781)
Immediate memory	- 0.001 (0.022, 0.992)	- 2.212 (0.001, 0.034)
Delayed Memory	- 0.009 (0.024, 0.900)	- 1.122 (0.001, 0.283)
Verbal Reasoning	- 0.010 (0.072, 0.899)	1.239 (0.003, 0.247)
Nonverbal reasoning	0.187 (0.016, 0.013)	- 0.191 (0.001, 0.857)
Language	0.061 (0.026, 0.423)	- 0.532 (0.001, 0.621)
g	0.043 (0.090, 0.555)	- 01.113 (0.004, 0.270)

## Discussion

We investigated the relationship between haemoglobin and cognition in a well-characterised cohort in late midlife. Our results highlight a linear relationship between haemoglobin levels and nonverbal reasoning scores and a quadratic relationship with immediate memory scores in which both high and low levels of haemoglobin are associated with reduced scores.

While our findings linking low haemoglobin with poorer cognition in older adults are consistent with the literature, there are some inconsistencies in this relationship within different domains of cognition. Our finding of a linear relationship between haemoglobin and nonverbal reasoning is somewhat consistent with a study by Qin et al. who found a reduction in Telephone Interview of Cognitive Status (TICS) scores in a large sample of older Chinese adults [8]. However, this association did not persist after adjusting for sociodemographic and health-related variables. It must be noted that TICS has not been extensively used for assessing nonverbal and visuospatial reasoning, with only a modified version having moderate association with these domains [17], whereas the Raven’s Progressive Matrices test employed in our study is a more robust measure of this [18]. Our finding displaying a quadratic relationship between haemoglobin and immediate memory is somewhat consistent with a study by Shah et al. who report a similar relationship between episodic memory in females but not all participants in their study [9]. They constructed episodic memory scores by combining scores of several tests including both immediate and delayed memory scores. However, they did not investigate these tests individually.

Other papers have found significant associations with bespoke scores for global cognition; Qin et al. found a linear relationship with a global cognitive score constructed by combining episodic memory and TICS scores [8], and Shah et al. found a quadratic relationship with a global cognitive score constructed by taking a centred average of 21 cognitive tests [9]. While we did not replicate this finding, ours is the only study, to the best of our knowledge, to use principal component analysis to construct a global cognitive score to investigate the association between haemoglobin and cognition. Other studies have reported a significant relationship with processing speed, semantic memory, attention, and mental status [5,9,19,20-21]. We did not find a significant relationship with processing speed and we did not measure semantic memory or attention. Other studies have replicated these findings using haemoglobin as a binary variable [3]; however, due to the low number of anaemic patients (n=8), we did not carry out this analysis. While most studies in the literature are cross-sectional in design, Qin et al. conducted longitudinal analysis and found that anaemia was linked with increased cognitive decline after a two-year follow-up in a population of ageing adults, strengthening a potential causal relationship [8]. Anaemia has also been extensively described as a predictor for Alzheimer disease in older adults [3]; however, this association has not been investigated longitudinally and an inverse relationship may be present wherein poorer cognition may result in individuals not meeting nutritional requirements. 

Due to the low number of anaemic males in our cohort (n=1), we did not assess the interactive effects of sex on these associations. One study reports that the relationship between haemoglobin and cognition was stronger in female participants [9] and another study reported an association between anaemia and reduced cognition amongst only male members of their study population [22]. Other studies have reported no significant interactive effect of sex in the association between haemoglobin and cognition [8]. The potential interactive effects of sex seem inconsistent but should be further investigated.

Several mechanisms have been proposed to explain the association between haemoglobin and cognition, the most prevalent suggesting that low levels of haemoglobin result in inadequate oxygen delivery to the brain. Low haemoglobin levels have been associated with impaired cerebral vascular regulation [23] and have been linked with neurological injury; a study by Park et al. found that decreased haemoglobin levels were linked with cortical atrophy in females [4]. Several studies have found associations between low haemoglobin and both reduced cognition and brain pathology within the same population thus strengthening the case for brain injury acting as a mechanism; Son et al. reported an association between anaemia, white matter hyperintensities, and cognition and noted an interactive effect between anaemia and white matter hyperintensities for one of their cognitive tests [6], and Jonassaint et al. found that lower haemoglobin levels were associated with poorer cognition along with reduced intracranial and grey matter volumes [5].

RPM is a classic test of nonverbal reasoning, commonly referred to as matrix reasoning, and has been used as a core component in deriving global cognition, *g* [24]. RPM assesses abstract, visual problem-solving ability without the language or motor demands of other cognitive assessments [15]. Participants are presented with geometric patterns in 2x2, 3x3, 4x4, or 6x6 matrices and are asked to identify the missing element that completes the pattern. RPM is a sensitive measure for cognitive sequelae of stroke and dementia [25]. Iodoamphetamine single-photon emission computed tomography (I-IMP SPECT) studies have shown that RPM scores are positively correlated with regional cerebral blood flow in various lobes [26]. Perhaps the impaired cerebral vascular regulation seen in those with low haemoglobin may explain this reduction in RPM scores.

Memory was assessed using the LM I and LM II subsections of the WMS-IV, which are robust and reliable assessments of this domain [15]. Immediate or short term memory describes an individual’s ability to hold but not manipulate recently presented information. Impaired memory is a classic and early feature of Alzheimer’s disease owing to the early hippocampal damage seen in the condition [27]. The hippocampus is particularly sensitive to dysregulation and is noted to have several microvascular differences that result in poorer cerebral blood flow homeostasis [28]. A recent study has demonstrated a U-shaped relationship between haemoglobin concentration and brain pathology in which both high and low haemoglobin concentrations are associated with a significant increase in white matter hyperintensity volume and a decrease in structural connectivity [29]. This may provide a mechanism explaining our quadratic finding with immediate memory.

The current study had numerous strengths. The first was the richness of the data; each participant was subject to an array of robust and sensitive cognitive tests testing several domains of cognition. Additionally, we were able to adjust for several important confounding variables, which solidified our analysis. To the best of our knowledge, we are the first study to use principal component analysis to construct a global cognitive score when conducting these analyses.

At the same time, there were several limitations with our study, most notable among which is the good health of our participants. Very few participants had abnormal haemoglobin levels, which meant that our analysis may have been underpowered to detect some significant associations. However, the presence of significant associations may suggest that even nonpathological changes in haemoglobin may impact the brain enough to lead to impaired cognition. We know that, in common with most cohort studies, our participants represent a healthier, wealthier, and better-educated sample compared with the general population from which they are drawn. Additionally, the cross-sectional design of our data prevents us from inferring causation. While we corrected for various covariates, we did not adjust for other confounders such as renal function and comorbidities, which have been included in other studies. However, given that this is a healthy sample, the confounding effects of these variables may be less significant.

Future work will involve longitudinal assessment of haemoglobin and cognition within the ACONF cohort in order to determine if this association is causal in nature. Additionally, magnetic resonance imaging data drawn from the STRADL group may offer further insights into the mechanism of action by which abnormal haemoglobin concentration led to decreased cognitive scores in this group.

The results of this study add to the growing literature on this association, help better characterise the relationship between haemoglobin and cognition, and supports larger studies of anaemia as a preventable risk factor for cognitive impairment and dementia.

## Conclusions

Haemoglobin concentration was found to have a significant linear association with nonverbal reasoning scores and a significant quadratic association with memory scores in a healthy cohort of older adults. While the limited number of participants with pathological haemoglobin may limit the strength of these associations, these findings add to the growing literature exploring the link between haemoglobin concentrations and cognition.

## References

[1] McLean E, Cogswell M, Egli I, Wojdyla D, de Benoist B (2009). Worldwide prevalence of anaemia, WHO Vitamin and Mineral Nutrition Information System, 1993-2005. Public Health Nutr.

[2] Joosten E, Detroyer E, Milisen K (2016). Effect of anaemia on hand grip strength, walking speed, functionality and 1 year mortality in older hospitalized patients. BMC Geriatr.

[3] Andro M, Le Squere P, Estivin S, Gentric A (2013). Anaemia and cognitive performances in the elderly: a systematic review. Eur J Neurol.

[4] Park SE, Kim H, Lee J (2016). Decreased hemoglobin levels, cerebral small-vessel disease, and cortical atrophy: among cognitively normal elderly women and men. Int Psychogeriatr.

[5] Jonassaint CR, Varma VR, Chuang YF, Harris GC, Yasar S, Polinder-Bos H, Carlson MC (2014). Lower hemoglobin is associated with poorer cognitive performance and smaller brain volume in older adults. J Am Geriatr Soc.

[6] Son SJ, Lee KS, Na DL (2012). The effect of anemia and white matter hyperintensities (WMH) on cognitive impairment in patients with amnestic mild cognitive impairment (MCI). Arch Gerontol Geriatr.

[7] Dlugaj M, Eisele L, Winkler A (2014). Anemia and mild cognitive impairment in a German general population. J Alzheimers Dis.

[8] Qin T, Yan M, Fu Z, Song Y, Lu W, Fu A, Yin P (2019). Association between anemia and cognitive decline among Chinese middle-aged and elderly: evidence from the China health and retirement longitudinal study. BMC Geriatr.

[9] Shah RC, Wilson RS, Tang Y, Dong X, Murray A, Bennett DA (2009). Relation of hemoglobin to level of cognitive function in older persons. Neuroepidemiology.

[10] Leon DA, Lawlor DA, Clark H, Macintyre S (2006). Cohort profile: the Aberdeen children of the 1950s study. Int J Epidemiol.

[11] Navrady LB, Wolters MK, MacIntyre DJ (2018). Cohort profile: stratifying resilience and depression longitudinally (STRADL): a questionnaire follow-up of Generation Scotland: Scottish Family Health Study (GS:SFHS). Int J Epidemiol.

[12] (2022). WHO: Haemoglobin concentrations for the diagnosis of anaemia and assessment of severity. http://www.who.int/vmnis/indicators/haemoglobin..

[13] (2022). Mayo Clinic: High hemoglobin count. http://www.mayoclinic.org/symptoms/high-haemoglobin-count/basics/definition/sym-20050862..

[14] Williams B, Mancia G, Spiering W (2018). 2018 ESC/ESH Guidelines for the management of arterial hypertension. Eur Heart J.

[15] Sherman EM, Tan JE, Hrabok M (2020). A compendium of neuropsychological tests: administration, norms, and commentary (3rd ed.). https://www.worldcat.org/title/compendium-of-neuropsychological-tests-administration-tests-norms-and-commentary/oclc/1175579389&referer=brief_results.

[16] Phelps RP (2009). Correcting fallacies about educational and psychological testing. https://www.apa.org/pubs/books/4318046.

[17] Crooks VC, Petitti DB, Robins SB, Buckwalter JG (2006). Cognitive domains associated with performance on the telephone interview for cognitive status-modified. Am J Alzheimers Dis Other Demen.

[18] Allen MD, Fong AK (2008). Clinical application of standardized cognitive assessment using fMRI. I. Matrix reasoning. Behav Neurol.

[19] Lucca U, Tettamanti M, Mosconi P (2008). Association of mild anemia with cognitive, functional, mood and quality of life outcomes in the elderly: the "Health and Anemia" study. PLoS One.

[20] Terekeci HM, Kucukardali Y, Onem Y (2010). Relationship between anaemia and cognitive functions in elderly people. Eur J Intern Med.

[21] Denny SD, Kuchibhatla MN, Cohen HJ (2006). Impact of anemia on mortality, cognition, and function in community-dwelling elderly. Am J Med.

[22] Argyriadou S, Vlachonikolis I, Melisopoulou H, Katachanakis K, Lionis C (2001). In what extent anemia coexists with cognitive impairment in elderly: a cross-sectional study in Greece. BMC Fam Pract.

[23] Gottesman RF, Sojkova J, Beason-Held LL, An Y, Longo DL, Ferrucci L, Resnick SM (2012). Patterns of regional cerebral blood flow associated with low hemoglobin in the Baltimore Longitudinal Study of Aging. J Gerontol A Biol Sci Med Sci.

[24] Jensen AR (2012). The "g" factor: the science of mental ability. Praeger: Westport, Conn.

[25] Ryan JJ, Carruthers CA, Miller LJ, Souheaver GT, Gontkovsky ST, Zehr MD (2005). The WASI matrix reasoning subtest: performance in traumatic brain injury, stroke, and dementia. Int J Neurosci.

[26] Yoshida T, Mori T, Shimizu H (2017). Neural basis of visual perception and reasoning ability in Alzheimer's disease: correlation between Raven's Colored Progressive Matrices test and 123 I-IMP SPECT imaging results. Int J Geriatr Psychiatry.

[27] Jahn H (2013). Memory loss in Alzheimer's disease. Dialogues Clin Neurosci.

[28] Shaw K, Bell L, Boyd K (2019). Hippocampus has lower oxygenation and weaker control of brain blood flow than cortex, due to microvascular differences [PREPRINT]. bioRxiv.

[29] Wolters FJ, Zonneveld HI, Licher S (2019). Hemoglobin and anemia in relation to dementia risk and accompanying changes on brain MRI. Neurology.

